# Corrected flow time and respirophasic variation in blood flow peak velocity of radial artery predict fluid responsiveness in gynecological surgical patients with mechanical ventilation

**DOI:** 10.1186/s12871-022-01837-9

**Published:** 2022-09-19

**Authors:** Jianjun Shen, Shaobing Dai, Xia Tao, Xinzhong Chen, Lili Xu

**Affiliations:** 1 Department of Anesthesiology, the Second Affiliated Hospital, School of Medicine, Zhejiang University, Hangzhou, China; 2 Department of Anesthesiology, Women’s Hospital, Zhejiang University School of Medicine, Hangzhou, Zhejiang Province China; 3 Department of Ultrasound, Women’s Hospital, Zhejiang University School of Medicine, Hangzhou, Zhejiang Province China

**Keywords:** Corrected flow time, Respirophasic variation in blood flow peak velocity, Radial artery, Ultrasonography, Fluid responsiveness, Gynecological

## Abstract

**Background:**

Recent evidence suggests that ultrasound measurements of carotid and brachial artery corrected flow time (FTc) and respirophasic variation in blood flow peak velocity (ΔVpeak) are valuable for predicting fluid responsiveness in mechanical ventilated patients. We performed the study to reveal the performance of ultrasonic measurements of radial artery FTc and ΔVpeak for predicting fluid responsiveness in mechanical ventilated patients undergoing gynecological surgery.

**Methods:**

A total of eighty mechanical ventilated patients were enrolled. Radial artery FTc and ΔVpeak, and non-invasive pulse pressure variation (PPV) were measured before and after fluid challenge. Fluid responsiveness was defined as an increase in stroke volume index (SVI) of 15% or more after the fluid challenge. Multivariate logistic regression analyses and receiver operating characteristic (ROC) curve were used to screen multivariate predictors of fluid responsiveness and identify the predictive abilitie of non-invasive PPV, ΔVpeak and FTc on fluid responsiveness.

**Results:**

Forty-four (55%) patients were fluid responders. Multivariate logistic regression analysis showed that radial artery FTc, ΔVpeak, and non-invasive PPV were the independent predictors of fluid responsiveness, with odds ratios of 1.152 [95% confidence interval (CI) 1.045 to 1.270], 0.581 (95% CI 0.403 to 0.839), and 0.361 (95% CI, 0.193 to 0.676), respectively. The area under the ROC curve of fluid responsiveness predicted by FTC was 0.802 (95% CI, 0.706–0.898), and ΔVpeak was 0.812 (95% CI, 0.091–0.286), which were comparable with non-invasive PPV (0.846, 95%CI, 0.070–0.238). The optimal cut-off values of FTc for fluid responsiveness was 336.6 ms (sensitivity of 75.3%; specificity of 75.9%), ΔVpeak was 14.2% (sensitivity of 88.2%; specificity of 67.9%). The grey zone for FTc was 313.5–336.6 ms and included 40 (50%) of the patients, ΔVpeak was 12.2–16.5% and included 37(46%) of the patients.

**Conclusions:**

Ultrasound measurement of radial artery FTc and ΔVpeak are the feasible and reliable methods for predicting fluid responsiveness in mechanically ventilated patients.

**Trial registration:**

The trial was registered at the Chinese Clinical Trial Registry (ChiCTR)(www.chictr.org), registration number ChiCTR2000040941.

**Supplementary Information:**

The online version contains supplementary material available at 10.1186/s12871-022-01837-9.

## Introduction

Perioperative volume management is an important part of clinical anesthesia work and is crucial to prevent postoperative complications and smooth recovery of patients. Insufficient infusion can cause low perfusion of heart, kidney, brain and other important organs, microcirculation disorder, and organ dysfunction, while excessive infusion can cause postoperative intra-abdominal hypertension, affect the recovery of gastrointestinal function after anastomotic healing, and increase the probability of systemic infection. In this context, a proper assessment of volume status, coupled with proper fluid management, can optimize the hemodynamics of patients and avoid ineffective or even harmful fluid infusion [1–3]. Over the past decades, a large body of evidence has showed that various dynamic parameters (both invasive and non-invasive) have emerged with a high sensitivity and specificity for predicting fluid responsiveness (FR) [4]. Among them, non-invasive pulse pressure variation (PPV) has been suggested as a simple indicator of liquid reactivity, as non-invasive and easy access [5, 6], however, it has also been reported that volume status cannot be accurately reflected in certain cases such as cardiac arrhythmias, increased intrathoracic or abdominal pressure, and reduced lung compliance [7, 8].

Recently, ultrasonic Doppler for measuring blood flow of superficial artery has been reported to be used in operation care settings due to its advantages of convenience, non-invasive, far away from the surgical field, and low technical requirements [9, 10]. Importantly, carotid artery corrected flow time (FTc) has been validated as an acceptable and regenerative method for the identification of fluid responsiveness in critically ill patients with undifferentiated shock [11]. Interestingly, after Kim et al. [12] confirmed that respirophasic variation in blood flow peak velocity (ΔVpeak) of carotid artery has the similar capability to predict fluid responsiveness in infants undergoing cardiac surgery, a systematic meta-analysis of Yao et al. [13] further described that ΔVpeak of carotid artery had more value than brachial artery in predicting fluid Responsiveness in mechanically ventilated patients. However, we still could not know that whether radial artery FTc and ΔVpeak could assess the effect of a fluid challenge and is ideally suited to guide fluid resuscitation in mechanically ventilated patients. Our aim was to compare the ability of ultrasonic measurements of radial artery FTc and ΔVpeak to predict fluid responsiveness with non-invasive PPV in mechanically ventilated patients undergoing gynecological surgery.

## Methods

### Patients

After approved by the Research Ethics Committee of Women’s Hospital, Zhejiang University School of Medicine (IRB-20200197-R), this study was registered at the Chinese Clinical Trial Registry (ChiCTR)(www.chictr.org) on 16/12/2020 with the registration number ChiCTR2000040941. All methods of the study was conducted in accordance with the Declaration of Helsinki after written informed consent was obtained from all patients. Eighty patients, American Society of Anesthesiologists (ASA) Class I-II, undergoing gynecological surgery under general anesthesia from 2020/12/14 to 2021/02/28 were recruited into the study. Exclusion criteria were pregnancy, ≤18 years of old, BMI > 30 or < 15 kg/m-2, patients receiving vasoactive or inotropic support before induction of anaesthesia, or with left ventricular ejection fraction (LVEF) less than 45%, pre-existing peripheral arterial occlusive disease, cardiovascular disease, hypertension, pulmonary hypertension, chronic lung disease, abnormal chest wall, atrial or ventricular arrhythmia, diabetes mellitus, cerebrovascular disease and so on.

### Anaesthetic management

Upon arrival in the operating theatre, a three-lead electrocardiogram (ECG), pulse oximetry (SpO_2_), and non-invasive arterial pressure monitoring were applied. After anesthesia induction of midazolam 0.04 mg/kg cisatracurium 0.2 mg/kg propofol 2 mg/kg sufentanil 0.5 μg/kg, endotracheal intubation was performed. Respiratory setting of anesthesia machine (Aestiva, GE/Datex-Ohmeda) are set as follows: volume-controlled ventilation (VCV), inspiratory-expiratory (I:E) ratio of 1:2, respiratory rate of 8–10 bpm, tidal volume of 8 mL/kg of ideal weight [45.5 + 0.91x (height in cm-152.4)], and PEEP of 5 cm H_2_O in 50% oxygen with air. Respiratory settings were adjusted to maintain the P_ET_CO_2_ at less than 50 mmHg. Anesthesia was maintained with continuous infusion of remifentanil (0.05–0.2 μg/kg^− 1^ min^− 1^), propofol (50-100 μg/kg^− 1^ min^− 1^), and sevoflurane (1.5–2.5%) and intermittent injection of cisatracurium 0.05 mg/kg as needed to keep the entropy scale between 40 and 60 and for muscle relaxation. The mean arterial pressure was keep between 60 and 80 mmHg.

### Study protocol

**Fig. 1:**
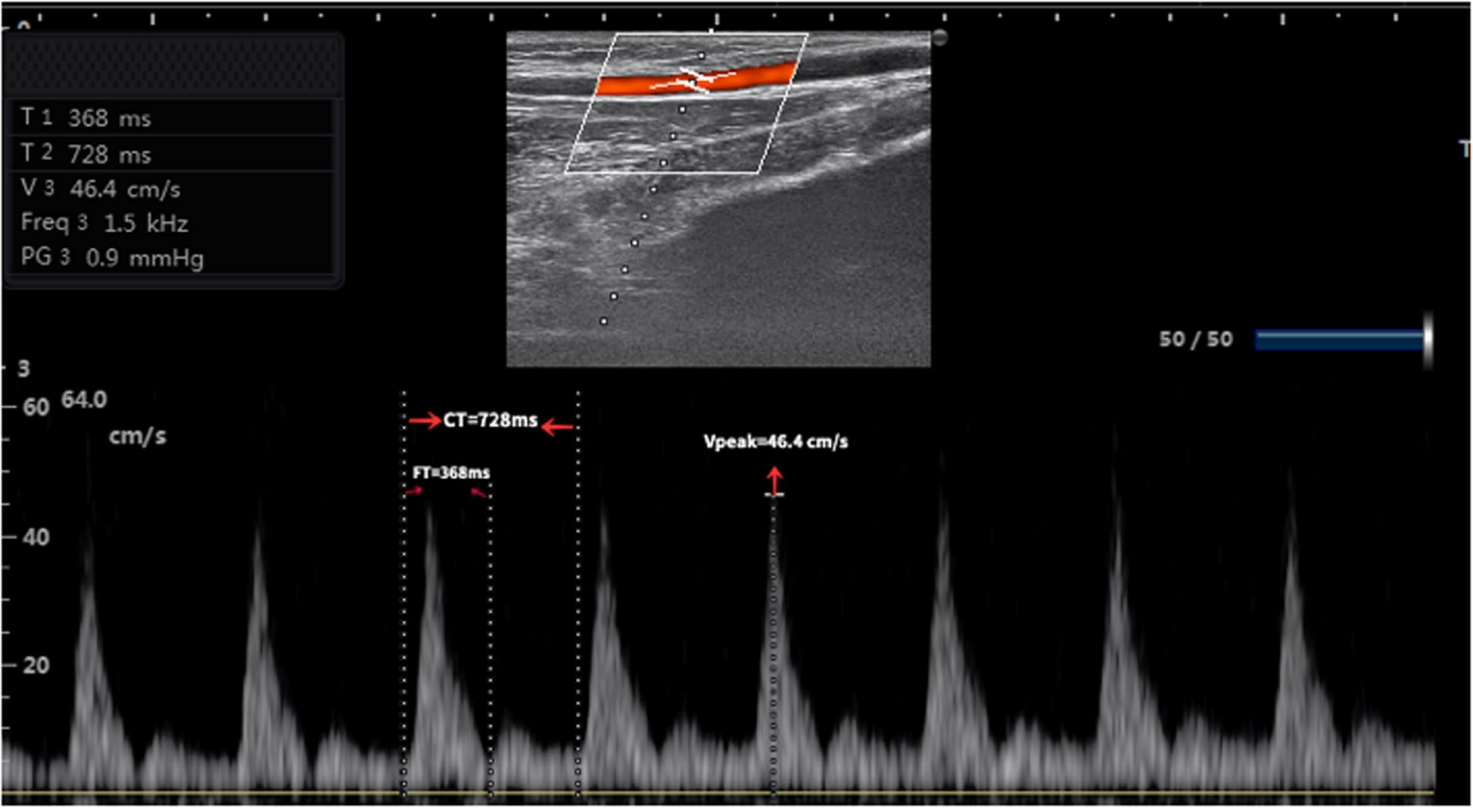
Example ultrasound images of radial artery FT and Vpeak. Radial artery FT and Vpeak were measured adjacent to the radial head. FTc was calculated by using a simplified formula FTc = FT+ [1.29 x (HR - 60)] [12]. ΔVpeak was calculated as follows: (maximum peak velocity-minimum peak velocity) / [(maximum peak velocity + minimum peak velocity)/2]× 100 [10]. FT: radial artery flow time; Vpeak: radial artery blood flow peak velocity

All measurements including the mean arterial pressure, heart rate, FTc and ΔVpeak of radial artery, non-invasive pulse pressure variation (PPV), and stroke volume index (SVI) were recorded 15 min after anesthesia induction and 10 min after a fluid loading of 6 ml/kg of 6% hydroxyethyl starch 130/0.4. The fluid challenge was performed over 10 min. All measurements were performed prior to the start of surgery. Non-invasive PPV was acquired from the right radial artery pressure waveform, using a real-time radial artery blood pressure and hemodynamics monitoring system (TL-400, Zhejiang Shanshi Biological Pharmaceutical Equipment Co., Ltd., Hangzhou, China). Averages of PPV auto over four cycles of 8 s were displayed in real time on the monitor and was calculated by automatic detection algorithms without airway pressure acquisition. The parameters were recorded by an anesthesiologist who was unaware of this study to avoid any personal bias. Rartery FTc and ΔVpeak consecutively measured by two independent sonographer who were blinded to each other’s Doppler results and haemodynamic variables of the patients, using an 6–13 MHz variable frequency linear probe ((SONIMAGE HS1, Konica Minolta Inc., Shanghai, China) (Fig. 1) and the order was randomized. The optimal long-axis view was gained at the left radial artery on the B-mode real-time image. The sampling site was located in the centre of the lumen, adjacent to the radial head and the ultrasonic beams were adjusted to ensure < 60 of angle from the direction of blood flow. The radial artery blood flow waveforms were stored using pulsed wave Doppler for the measurement of FTc and ΔVpeak. FT is measured from the beginning of the upstroke to the trough of the incisural notch on a pulse waveform analysis. FTc was calculated by using a simplified formula: FTc = FT+[1.29x (HR-60)] [14] as evaluating a single cycle after several consecutive cycles became stable and reached the level of acceptable quality. The maximum and minimum values of the peak velocity during one respiratory cycle were measured automatically and recorded. ΔVpeak was calculated as follows: (max peak velocity-min peak velocity)/[(max peak velocity + min peak velocity)/2] × 100 [15]. SVI was recorded by transthoracic echocardiography with a 1.5–4.5 MHz phased array probe from aortic blood flow. The diameter of the left ventricular outflow tract was determined by using the ultrasound images of the largest opening of the aortic valve on the parasternal long-axis view and the left ventricular outflow tract area was calculated as π x (left ventricular outflow tract diameter/2) [16]. The aortic flow time velocity integral was obtained in the apical five-chamber view at the level of the aortic annulus and from the mean of five consecutive beats of a complete respiratory cycle. SVI and BSA were computed by two formulas as follows: (left ventricular out-flow tract area x aortic flow time velocity integral)/body surface area (BSA), BSA (m2) = 0.0061x body length (cm) + 0.0128 x body weight (kg)-0.1529 [17]. All values represented the mean of three consecutive measurements and the mean of the two sonographer was employed for analysis.

### Study endpoints

The primary endpoint was to determine the predictive value of FTc, ΔVpeak, and non-invasive PPV for fluid responsiveness (≥15% increases in SVI after fluid challenge) in mechanically ventilated patients [18].

### Statistical analysis

SPSS 23 (SPSS Inc., Chicago, IL, USA) and PASS 14.0.5 (NCSS Statistical Software, Kaysville, UT, USA) were applied for statistical analysis and calculating the sample size. It has been reported that the area under the receiver operating characteristic (AUROC) curve of FTc measured in the radial artery to predict fluid responsiveness was 0.84 [19], so we assumed that the AUROC curve of radial artery FTc was 0.75. At least 42 patients were required to detect a difference of 0.25 between the AUROC curves of radial artery FTc (0.75) and non-invasive PPV (0.5), with an 0.9 power and type I error of 0.05, assuming 55% incidence of fluid responsiveness in patients undergoing elective gynecological surgery [9]. We used a sample size of 46 patients considering a possible 10% drop out rate. Fluid responsiveness was determined by a 15% or more increase in SVI after fluid challenge.

We evaluated the normal distribution of the data using Shapiroe-Wilk and Kolmogorove-Smirnov tests. If the data were normally distributed, continuous variables were expressed by mean (standard deviation), otherwise by median (interquartile range). Categorical variables are represented by absolute numbers (%). Paired t-test, ManneWhiney U-test, and X^2^ test were used for normally distributed data, non-normally distributed data, and categorical variables, respectively. Moreover, we used pearson correlation coefficient to detect the relationship between percentage change in SVI and relative changes in haemodynamic variables during fluid challenge. We also performed multivariate logistic regression analyses to screen multivariate predictors of fluid responsiveness and receiver operating characteristic (ROC) curve to identify the abilities of non-invasive PPV, ΔVpeak and FTc to predict fluid responsiveness. Furthermore, we defined the predictive accuracy of the ROC analysis as excellent [area under the curve (AUC) 0.9–1.0], good (AUC 0.8–0.9), fair (AUC 0.7–0.8), and poor 0.6–0.7 (AUC), calculated the 95% confidence interval (CI), and accepted statistical significance as p < 0.05 [20]. A DeLong test was performed to compare the three ROC curves as the previous study [21]. We also assessed the “optimal” cut-off values by maximizing Youden’s index (J = Sensitivity+Specificity-1 = Sensitivity-False-Positive Rate) [22] and determined the gray area by a correlation value of 90% specificity and 90% sensitivity [23]. Importantly, inter-observer variability (reproducibility) and the intra-observer variability (repeatability) of FTc and ΔVpeak were tested by dividing the absolute difference between the two values by their average value. Accordingly, the inter-observer reproducibility for ΔVpeak and FTc was validated by an intraclass correlation coefficient (ICC) and a coefficient of variation (CV) and Blande-Altman plot was used to test the inter-observer agreement.

## Results

### Patients

Of the 85 patients assessed for eligibility, 5 were excluded because of history of cardiovascular disease (*n* = 1), refusal to participate (*n* = 2), and other reasons (*n* = 2). Therefore, 80 subjects were enrolled in the final analysis (Supplementary Fig. 1). The main characteristics of the subjects were comparable between responders (*n* = 44) and non-responders (*n* = 36) (Table 1).

**Table 1:** Patient characteristics

	Responders group (***n*** = 44)	Non-responders group (***n*** = 36)	***P*** value
Age (yr)	33.3 ± 4.9	31.3 ± 5.6	0.090
ASA (I/II)	44/0	33/3	0.087
Height (cm)	160.0 ± 5.5	161.2 ± 5.6	0.321
Weight (kg)	55.4 ± 6.7	59.0 ± 8.9	0.054
BMI	21.7 ± 2.2	22.7 ± 3.1	0.096
Duration of Surgery (min)	108.4 ± 60.5	93.3 ± 31.1	0.154

*BMI* Body mass index (kg/m^2^), *HR* Heart rate, *MAP* Mean arterial pressure

Values are numbers or means±SD

*p < 0.05 compared with Responders group

### Haemodynamic variables before and after fluid challenge

**Fig. 2:**
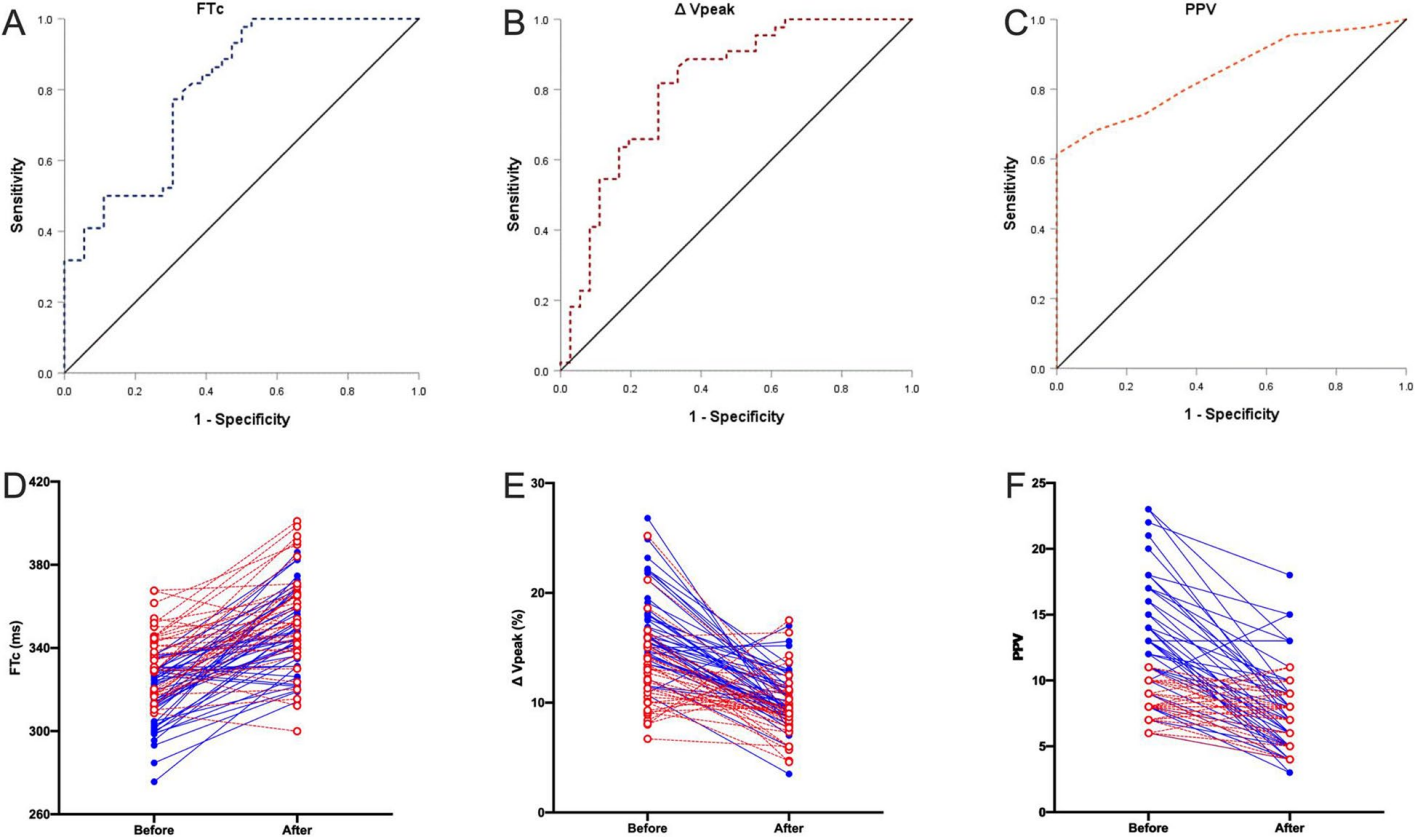
Individual responses to fluid challenge and ROC curve for FTc, ΔVpeak and PPV. Upper row: individual responses to fluid challenge for FTc (**A**), ΔVpeak (**B**) and PPV (**C**). Responders are presented as blue full line and closed circles; Non-responders are presented as red dashed line and open circles. Lower row: receiver operating characteristic curves showing the ability of FTc (**D**), ΔVpeak (**E**) and PPV (**F**) before fluid challenge to discriminate responders and non-responders. The areas under the curves for FTc, ΔVpeak and PPV were 0.802 (95% confidence interval 0.706–0.898), 0.812 (95% confidence interval 0.714–0.909), and 0.846 (95% confidence interval 0.762–0.930), respectively. FTc: radial artery corrected flow time; ΔVpeak: respirophasic variation in radial artery blood flow peak velocity; PPV: pulse pressure variation

In both responders and non-responders, fluid challenge significantly increased FTc and SVI, while significantly decreased ΔVpeak and non-invasive PPV(*p* < 0.05) (Table 2) (Fig. 2). Before the fluid challenge, FTc and SVI were significantly lower in responders than in non-responders (*p* < 0.05), however, ΔVpeak and non-invasive PPV was significantly higher in responders than in non-responders (*p* < 0.05) (Table 2). In contrast, after fluid challenge, FTc, ΔVpeak, and SVI were all not significantly different between the two groups (Table 2). Both MAP and HR were not significantly different between the two groups before and after the fluid challenge (Table 2).

**Table 2:** Hemodynamic variables before and after fluid challenge

	Responders group (***n*** = 44)		Non-responders group (***n*** = 36)		***P*** value	***P*** value
	Before	After	Before	After	Before	After
**FT**_**C**_ **(ms)**	**315.9 ± 15.0**	348.2 ± 19.0*	**335.1 ± 16.2#**	354.7 ± 24.5*	**0.000000**	**0.184**
**ΔVpeak (%)**	**16.8 ± 3.7**	10.4 ± 2.5*	**12.7 ± 3.9#**	9.4 ± 2.9*	**0.000007**	**0.136**
**PPV (%)**	**13.2 ± 4.5**	7.4 ± 3.6*	**8.3 ± 1.5#**	7.4 ± 2.0*	**0.000000**	**0.957**
**SVI (ml m**^**−2**^**)**	**30.0 ± 5.9**	39.7 ± 8.2*	**36.1 ± 6.8#**	**38.7 ± 6.9**	**0.000018**	**0.545**
**MAP (mmHg)**	**72.9 ± 12.4**	77.8 ± 13.2*	**75.0 ± 9.6**	**75.1 ± 8.6**	**0.411**	**0.301**
**HR** **(beat min-1)**	**73.8 ± 13.5**	64.1 ± 9.8*	**71.0 ± 13.5**	60.9 ± 8.9*	**0.356**	**0.134**

*FT*_*C*_ radial artery corrected flow time, *ΔVpeak* respirophasic variation in radial artery blood flow peak velocity, *PPV* Pulse pressure variation, *SVI* Stroke volume index

Data are reported as mean ± SD

*p < 0.05 compared with before fluid challenge*.* #*p* < 0.05 compared with Responders group

### The ability of FTc and ΔVpeak to predict fluid responsiveness

FTc, ΔVpeak, and non-invasive PPV were proved to be the independent predictors for fluid responsiveness by multivariate logistic regression, with the odds ratios of 1.152(95% CI 1.045 to 1.270), 0.581 (95% CI 0.403 to 0.839), and 0.361 (95% CI, 0.193 to 0.676), respectively (Table 3). The regression equation for predicting fluid responsiveness in pregnant women is logit *P* = -28.153 + 0.142 FTc − 0.543ΔVpeak-1.018 PPV. The area under the ROC curve of fluid responsiveness predicted by FTC was 0.802 (95% CI, 0.706–0.898), and ΔVpeak was 0.812 (95% CI, 0.091–0.286), which were comparable with non-invasive PPV (0.846, 95%CI, 0.070–0.238) (Table 4). The sensitivity and specificity for FTc and Vpeak are 75.3, 75.9 and 88.2%, 67.9% (Table 4). Their cut-off values for FTc and Vpeak are 336.6 ms and 14.2% (Table 4).

**Table 3:** Multivariate logistic regression analyses identified the factors that were independently associated with fluid responsiveness

	***B*** value	***P*** value	Odds ratio (95% CI)
FTC (ms)	0.142	0.004	1.152 (1.045–1.270)
ΔVpeak (%)	-0.543	0.004	0.581 (0.403–0.839)
PPV (%)	-1.018	0.001	0.361 (0.193–0.676)

*FT*_*C*_ radial artery corrected flow time; *ΔVpeak* Respirophasic variation in radial artery blood flow peak velocity, *PPV* Pulse pressure variation

**Table 4:** Prediction of fluid responsiveness by receiver operating characteristic curves of the baseline FTc, ΔVpeak and PPV

	AUROC curve (95% CI)	***P***-value	Optimal cut-off value	Grey zone	Patients in grey zone (%)	Sensitivity (95% CI)	Specificity (95% CI)	Youden index (95% CI)
FTc	0.802 (0.706–0.898)	0.0004	336.6 ms^a^	313.5–336.6 ms	40 (50%)	0.75 (0.66–0.85)	0.76 (0.71–1.00)	0.477
ΔVpeak	0.812 (0.714–0.909)	0.0002	14.2%^a^	12.2–16.5%	37 (46%)	0.88 (0.79–0.97)	0.68 (0.60–0.77)	0.540
PPV	0.846 (0.762–0.930)	0.0001	11.5%^a^	7.5–11.5%	48 (60%)	0.74 (0.55–0.93)	0.54 (0.46–0.63)	0.614

*AUROC* Area under the receiver operating characteristic, *CI* Confidence interval, *FTC* Radial artery corrected flow time, *ΔVpeak* respirophasic variation in radial artery blood flow peak velocity, *PPV* Pulse pressure variation

^a^ Optimal cut-off values were determined by maximising the Youden index

### The inter-observer agreement in estimating FTc and ΔVpeak

For FTc measurements, intra-observer variability and inter-observer variability were 0.5 (0.3)% and 1.1 (0.9)%, respectively. For ΔVpeak measurements, inter-observer variability was 7.0 (9.3)% and 5.6 (2.8)%, respectively. Inter-observer reproducibility for estimating FTc was excellent, with an ICC of 0.97 (95% CI, 0.948–0.984) and a CV of 5.6%. Inter-observer reproducibility for estimating ΔVpeak was also excellent, with an ICC of 0.98 (95% CI, 0.973–0.988) and a CV of 28.7%. Using Bland-Altman analysis for evaluating inter-observer agreement in estimating FTc and ΔVpeak, the mean biases were − 0.26 ms [with 95% limits of agreement (LOA) between − 9.34 and 8.82 ms] and 0.41% (with 95% LOA between − 1.19 and 2.00%), respectively (Supplementary Fig. 2).

## Discussion

In our study, we performed the study to determine the ability of ultrasonic measurements of radial artery FTc and ΔVpeak for predicting fluid responsiveness and found that the two indices were the independent predictors of fluid responsiveness and the area under the ROC curve of fluid responsiveness predicted by FTC was 0.802 (95% CI, 0.706–0.898), and ΔVpeak was 0.812 (95% CI, 0.091–0.286). These results demonstrated that the two parameters assessed by Doppler ultrasound were valid and reliable predictor for determining fluid responsiveness in mechanical ventilated patients undergoing gynecological surgery.

Recently, ultrasonic Doppler for measuring blood flow of superficial artery has been proved to be successful in predicting the fluid responsiveness [9, 10]. FTc is a complex static index and has been used and evaluated as a preload indication to predict fluid responsiveness in different surgical settings and the use of FTc for intraoperative volume optimization has been reported to reduce the incidence of complications, improve patients’ recovery, and decrease postoperative hospital stay [24, 25]. It has been confirmed that it is affected by various factors such as inotropic state, afterload and preload, and is negatively correlated with systemic vascular resistance and post-load [26]. However, low FTc does not always correspond to low left ventricular preload and can even represent a volume overload state, which means that simple fluid challenge guided by only FTc could further aggravate deterioration in haemodynamic conditions [27]. We therefore tested the predictive accuracy of FTc to discriminate between responders and non-responders according to a volume load during gynecological surgery compared with non-invasive PPV. Based on our findings, FTc and non-invasive PPV accurately predicted FR attributable to both volume-loading manoeuvres, indicating interchangeability of these variables in this specific patient population. In this context, ROC analysis yielded similar levels of AUCs for non-invasive approaches during fluid resuscitation in the OR. With respect to statistical comparison of the ascertained AUC values, we found that FTc can predict fluid response in mechanical ventilated patient and no significant differences between FTc and non-invasive PPV. However, our findings appear to contradict those of two previous studies [28, 29], where FTc was not a predictor of fluid responsiveness. It is possible that patients with haemodynamic conditions that would prevent FTc from predicting fluid responsiveness were not excluded and vasoconstriction by norepinephrine may cause low FTc regardless of left ventricular preload state, which could be why FTc failed to predict fluid responsiveness in both studies. Instead, several recent studies strongly confirmed our results. For example, Lee et al. [9] demonstrated that FTc and PPV are better than CVP and LVEDAI in predicting fluid responsiveness in neurosurgical patients. Yang et al. [30] both FTc and PPVauto were accurate predictors of fluid responsiveness in patients in the supine position and the prone position using a Wilson frame undergoing lumbar spine surgery. In addition, MAITRA et al. [19] addressed that pressure transducer derived radial artery cFT correlated with Doppler derived carotid artery FTc and may be a reasonable predictor of volume responsiveness. In this context, FTc can be used to evaluate the effect of the treatments administered or can be integrated as a limit to optimize CO while avoiding excessive fluid loading. However, there may be no single parameter that can guide fluid therapy under all situations, FTc may be extremely useful when interpreted in conjunction with other clinical information, and measurements such as non-invasive PPV.

Up to now, numerous studies have been conducted to determine the ability of ΔVpeak to predict fluid responsiveness and its cut-off value in discriminating between responders and non-responders to fluid resuscitation [15, 31, 32]. Accordingly, the ΔVpeak of aortic artery, carotid artery and brachial artery have been successively prove to be the accurate method of evaluating preload and the promising variable shown to predict fluid responsiveness in ventilated surgical patients, critically ill patients or different kinds of shock [12, 15, 33]. Of note, the finding of Song et al. [15] confirmed that the ΔVpeak of carotid artery was the most appropriate for prediction of fluid responsiveness when compared with the invasive and noninvasive dynamic variables derived from the arterial pressure and the plethysmographic waveforms in mechanically ventilated patients undergoing coronary revascularization. Suggested cut-off values of ΔVpeak are 11% [15], which is possibly attributable to the variations in study population, such as surgical patients without concomitant disease or critically ill patients. In the same context, the radial artery is peripheral artery and also provides easy accessibility [32]. The radial artery ΔVpeak as determined by ultrasonic Doppler is a non-invasive and practical bedside monitor, there might be a role for radial artery ΔVpeak as a predictor of fluid responsiveness in certain clinical situations [32]. Based on these theoretical advantages, we investigated the feasibility and predictive power of Doppler-acquired respirophasic radial flow dynamics on fluid responsiveness in mechanically ventilated patients. As our results indicated, the predictability of radial artery ΔVpeak was comparable to that of non-invasive PPV with excellent interobserver agreement. Moreover, radial artery ΔVpeak yielded a cut-off value with the highest sensitivity and specificity. Interestingly, we also found that radial artery ΔVpeak also showed a significant increase in non-responders after the fluid challenge, suggesting its strong association with preload, while non-invasive PPV showed a significant decrease after fluid challenge even in non-responders. However, as previous study mentioned, the most commonly accessed radial artery could yield erroneous information regarding systemic vascular resistance and respirophasic variations in stroke volume as well the PPV (or SVV) obtained from the radial artery would yield inconclusive or inaccurate information [15, 34]. In the present study, we comprehensively evaluated the ability of the radial artery to predict volume responsiveness from both the respiratory variability of the radial artery pressure and blood flow, which can provide more favorable evidence for the clinical use of the radial artery in evaluating volume state. Thus, it remains to be verified through further studies and more clinical experience.

Among the dynamic indices, PPV originated from arterial waveform analysis is the representative indices for fluid responsiveness and reflects the percentage change in pulse pressure attributable to periodic changes in intrathoracic pressure caused by mechanical ventilation [26]. Colquhoun et al. found the agreement between estimates of the Tensys T-line and an intra-arterial catheter (for both mean arterial pressure [MAP] and pulse pressure variation [PPV]) in the setting of spine surgery [5]. Based on the premise that non-invasive and readily accessed indices are undoubtedly advantageous, non-invasive arterial pressure measurement based on radial artery tonometry using the TL-200pro technology is capable of providing MA*P* values with high accuracy (low mean difference) and precision (narrow limits of agreement) and is feasible in medical ICU patients [6]. In general, our results showed that non-invasive PPV were predictive of fluid responsiveness in mechanical ventilated patients undergoing gynecological surgery, with the area under the ROC curve of 0.846 (95%CI, 0.762–0.930), a cut-off value of 11.5%, and a grey zone between 7.5 and 11.5%, which are in accordance with the above-mentioned study. However, several exiting factors including technical factors, arterial compliance, cardiac arrhythmias, increased intrathoracic pressure by large tidal volume or PEEP, increased abdominal pressure, and reduced lung compliance restrict its widespread use during surgery [7, 8].

There are several limitations in our study. First, we did not study the ability of FTc and ΔVpeak of radial artery and non-invasive PPV to predict fluid responsiveness in patients during persistent hypotension, hypothermia, septic shock, heart failure, significant valvular heart diseases, or significant radial artery stenosis, therefore, our results cannot be extrapolated to these patients and the generalization of these results may be limited. Further researches are needed to more clearly identify the confounders in order to determine the limitations and indications of these non-invasive assessments of fluid responsiveness. Second, as other dynamic indices based on heart-lung interactions, FTc and ΔVpeak of radial artery and non-invasive PPV have their limitations and could not be used in patients with cardiac arrhythmias or spontaneous breathing. Nevertheless, as previous studies reported [35], few studies have been conducted to determine the ability of non-invasive indices to predict fluid responsiveness and its cut-off value in discriminating between responders and non-responders to fluid resuscitation in the spontaneously breathing or cardiac arrhythmias patient. Consequently, there is a clear need for a reliable non-invasive method for the assessment of volume status and fluid responsiveness in these patient population and clinical setting. Third, FTc and ΔVpeak might be still reliable in patients with decreased arterial compliance, when the predictive power of non-invasive PPV for fluid responsiveness is reduced. Further studies are needed to test their performance in haemodynamically unstable patients under low perfusion status.

In conclusion, the principal finding of this study is that the measures of FTc and ΔVpeak in the radial artery assessed by Doppler ulrasound appears to be the highly feasible and reliable methods to predict fluid responsiveness, which are valuable and interchangeable with non-invasive PPV in patients undergoing gynecological surgery. Thereby, these results suggested that a non-invasive approach using the dynamic variables of fluid responsiveness in order to maintain or to achieve euvolaemia could serve as useful indices to guide fluid therapy during gynecological surgery. Nevertheless, there may be no single index that can guide fluid therapy in all cases, so every clinical finding and all haemodynamic data should be applied when needed. Combining FTc, ΔVpeak, and non-invasive PPV can be used to predict fluid responsiveness in gynecologic surgical patients and the regression equation for predicting fluid responsiveness is logit *P* = -28.153 + 0.142FTc-0.543ΔVpeak-1.018PPV. In the future, more clinical investigations and application experience remained to furtherly illuminate and verify their ability for predicting fluid responsiveness and guiding clinic fluid therapy.

## Supplementary Information

**Additional file 1: Supplementary Fig. 1.** Subject selection process.

**Additional file 2: Supplementary Fig. 2.** BlandeAltman plots for inter-observer agreement of radial artery FTc and ΔVpeak.

## Data Availability

The datasets generated and/or analysed during the current study are not publicly available because the study has not been officially published but are available from the corresponding author on reasonable request.
