# Supplementary figures and images for: Corrected flow time and respirophasic variation in blood flow peak velocity of radial artery predict fluid responsiveness in gynecological surgical patients with mechanical ventilation
Source: BMC Anesthesiol. 2022 Sep 19;22:299. doi: 10.1186/s12871-022-01837-9 (PMC9484168; doi:10.1186/s12871-022-01837-9)

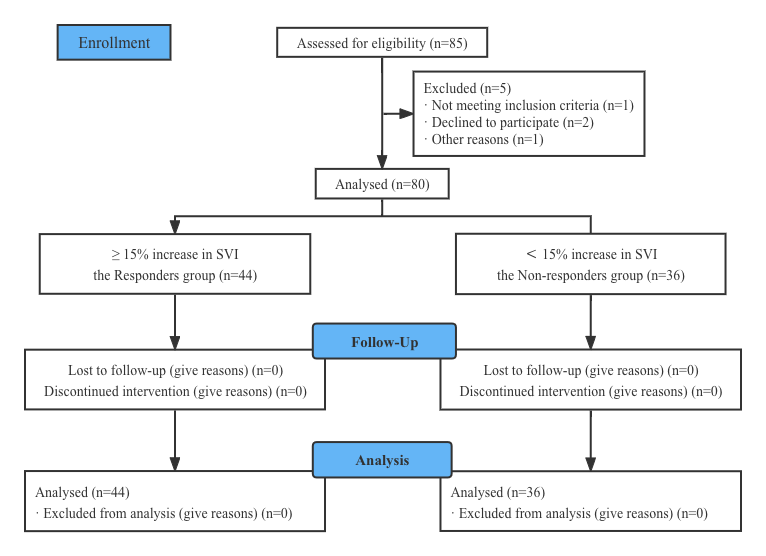

Supplement: Supplementary file 1 — Additional file 1: Supplementary Fig. 1. Subject selection process. [file 12871_2022_1837_MOESM1_ESM.png]

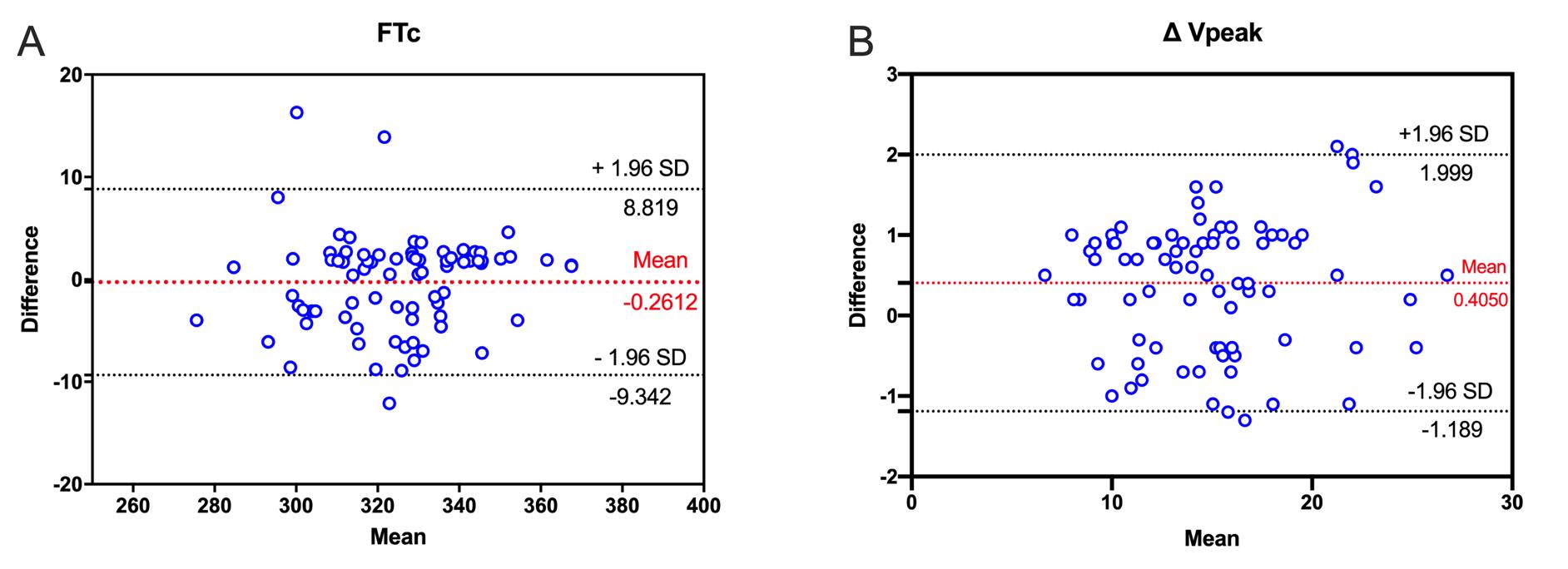

Supplement: Supplementary file 2 — Additional file 2: Supplementary Fig. 2. BlandeAltman plots for inter-observer agreement of radial artery FTc and ΔVpeak. [file 12871_2022_1837_MOESM2_ESM.jpg]
